# Relationship between Objectively Measured Walkability and Exercise Walking among Adults with Diabetes

**DOI:** 10.1155/2014/542123

**Published:** 2014-03-26

**Authors:** Akiko S. Hosler, Mary P. Gallant, Mary Riley-Jacome, Deepa T. Rajulu

**Affiliations:** ^1^Department of Epidemiology and Biostatistics, University at Albany School of Public Health, One University Place, Rensselaer, NY 12144, USA; ^2^Department of Health Policy, Management and Behavior, University at Albany School of Public Health, One University Place, Rensselaer, NY 12144, USA; ^3^New York New Jersey Preparedness and Emergency Response Learning Center, University at Albany School of Public Health, One University Place, Rensselaer, NY 12144, USA; ^4^AIDS Institute, New York State Department of Health, Corning Tower, Empire State Plaza, Albany, NY 12222, USA

## Abstract

Little is known about the relationship between objectively measured walkability and walking for exercise among adults with diabetes. Information regarding walking behavior of adults with diabetes residing in 3 Upstate New York counties was collected through an interview survey. Walkability measures were collected through an environmental audit of a sample of street segments. Overall walkability and 4 subgroup measures of walkability were aggregated at the ZIP level. Multivariate logistic regression was used for analysis. Study participants (*n* = 208) were 61.0% female, 56.7% non-Hispanic White, and 35.1% African-American, with a mean age of 62.0 years. 108 participants (51.9%) walked for exercise on community streets, and 62 (29.8%) met the expert-recommended level of walking for ≥150 minutes/week. After adjustment for age, gender, race/ethnicity, education, BMI, physical impairment, and social support for exercise, walking any minutes/week was associated with traffic safety (OR 1.34, 95% CI 1.15–1.65). Walking ≥150 minutes/week was associated with overall walkability of the community (2.65, 1.22, and 5.74), as well as sidewalks (1.73, 1.12–2.67), street amenity (2.04, 1.12–3.71), and traffic safety (1.92, 1.02–3.72). This study suggests that walkability of the community should be an integral part of the socioecologic approach to increase physical activity among adults with diabetes.

## 1. Introduction

Walking is a low-impact, moderate-intensity aerobic physical activity that requires little equipment or training. It is the most popular physical activity among Americans of various sociodemographic backgrounds and physical abilities, including individuals with diabetes [1–4]. Research has found that walking has several health benefits to adults with diabetes. Meta-analyses indicate that routine walking and other types of moderate aerobic exercise can improve glycemic control as indicated by decreased HbA1c [5, 6] and lower systolic blood pressure, triglycerides, and waist circumference [7]. Routine walking is also independently associated with lower levels of circulating proinflammatory markers [8], significant reduction of cardiovascular events [9], and lower cardiovascular mortality and all-cause mortality in adults with diabetes [10, 11].

Most Americans use community streets for leisure time walking [12]. Walking on community streets is convenient and inexpensive and can be sustainable. When planning to promote community walking, however, one should consider environmental barriers and enablers for walking in the community. A volume of literature indicates that communities with highly walkable physical environments are associated with a higher probability of walking [13–16]. For instance, the walkability of a community is a better predictor of daily physical activity than community income [14], and people living in high walkability communities have 28% higher odds of walking for exercise compared to those in low walkability communities [15].

It is important to separate walking for transport (destination walking) and walking for exercise, because these are two of different constructs and influenced by different sets of environmental factors [17]. Furthermore, the effects of the environment on walking behavior can be modified by personal factors such as age, gender, race/ethnicity, and physical ability [18]. Diabetes status is an important personal factor to consider, as adults with diabetes are more likely to be older and obese [19] and have lower extremity complications [20] that could cause or increase pain during physical activity [21]. Functional impairment [22] and vision impairment [23] are also common in older adults with diabetes. Therefore adults with diabetes are likely to be sensitive to environmental features in their decision to walk for exercise.

Only a handful of studies have examined the relationship between the built environment and walking or physical activity specific to adults with diabetes, and they found such factors as perceived availability of walkable streets, places to visits, and aesthetic of the neighborhoods as environmental correlates of walking [24–26]. All these existing studies, however, relied on perceptions as measures of the environment. While perceived environments are recognized indicators of physical activity in adults with diabetes [27], these subjective measures can be poor proxies of objectively measured environments [28]. When planning interventions to improve the built environment, objective measures are useful to target and prioritize specific environmental features.

The objective of this study is to examine the cross-sectional association between objectively measured walkability in the community and walking for exercise (any minutes/week and ≥150 minutes/week) on community streets while controlling for known correlates.

## 2. Materials and Methods 

### 2.1. Study Setting

The setting of this study was the downtown portion of the city of Albany and the entire Columbia and Greene Counties in Upstate New York. Through the analyses of the population census and hospital coverage data, these communities were identified as medically underserved communities within the catchment area of our research center [29].

### 2.2. Study Design and Sample

This study employed a cross-sectional design and used two data sources. The first data source was a diabetes patient interview survey. Convenience sampling with quotas (based on population size per geographic region) was used to recruit participants. Criteria for participation included residing in one of the study communities, being 18 years of age or older, having been diagnosed with diabetes by a healthcare professional, and being able to understand the written consent. Participants were recruited at community locations including senior centers, housing complexes, churches, community fairs, flu vaccination clinics, soup kitchens, and health centers. They were interviewed in person by a trained survey taker who used a structured questionnaire written in English and Spanish. A total of 208 adults with diabetes completed the survey. The University at Albany Institutional Review Board reviewed and approved all study protocols.

The second data source is a walkability audit study. A stratified random sampling method was used to select a sample of street segments. The Census Block Groups (CBGs) that fell in the study communities were grouped into urban (*n* = 60), suburban (*n* = 22), and rural (*n* = 64) strata based on the census' place designations. One street segment was randomly selected from each of the urban and suburban CBGs, except for 5 large urban CBGs where street segments were oversampled with the rate of 2 streets per CBG (*n* = 4) and 3 streets per CBG (*n* = 1). For rural CBGs where there were very little within-group variations, 22 CBGs were randomly selected, and then one street segment was randomly chosen from each of the selected CBGs. A short walkability measurement tool called the Walking Environment Audit Tool-Diabetes (WEAT-D) was developed for this study. It contains 20 street environment indicators that are relevant to adults with diabetes, easily identifiable, and have a range of variations within the study communities. A team of trained surveyors audited the sampled street segments on foot on fair-weather days. Spatial positions of the street segments were measured using a handheld GPS unit. Physical measurements of street structures were taken manually using an industrial tape measure. A total of 110 street segments were in the sample, and all of them were successfully audited.

### 2.3. Measurements

#### 2.3.1. Individual-Level Measures

Levels of walking for exercise were measured by a series of questions asking respondents about the types, frequencies, durations, and locations of physical activity, which are adopted from the Behavioral Risk Factor Surveillance System questionnaire [30]. Respondents were classified as community exercise walkers when they had all of the following conditions: engaged in any physical activity during the previous month; identified walking as their primary or secondary physical activity; and used outdoors in their communities as places of walking or exercise. Approximate minutes spent for exercise walking per week were obtained by combining the reported frequencies (times per week or month) and duration (minutes or hours usually spent at a time) of this activity. In this study, walking for exercise in the community for any total length of time is called as “any walking.” A subset that involved walking 150 minutes/week or longer is identified as “recommended-level walking,” since the expert panel of the American Diabetes Association recommends moderate aerobic physical activity for at least 150 minutes/week to achieve glycemic control [31].

Several individual-level covariates that can influence walking behavior were also measured. Physical impairment was measured by the question “do you have any physical challenges that limit your ability to be physically active?” Body mass index (BMI) was calculated by self-reported height without shoes and weight that was marked off on a card and handed to the interviewer in an envelope. Social support for exercise was obtained from the modified Chronic Illness Resource Survey questions, which measured the level of social support from “not at all” (1 point) to “a great deal” (5 points) [32]. A composite variable indicating the highest level of social support for exercise provided by friends, spouse/partner, or children was created. Age, race/ethnicity, and educational attainment were self-reported by the respondent, and gender was assessed by the interviewer.

#### 2.3.2. Walkability Measures

Each of 20 walkability indicators was assigned with a numeric score that ranged from 1 to 4, with 4 representing the most desirable feature (Table 1). The scoring system was developed based on relevant expert recommendations, including those for sidewalk and street designs for older adults and people with disabilities supported by the Federal Highway Administration [33] and the Institute of Transportation Engineers [34]. The overall walkability measure was represented by the unweighted average of all 20 single-item scores. Subgroup measures were calculated by averaging items belonging to the following 4 dimensions: “sidewalks” (9 measures of sidewalk coverage, design, and materials), “traffic safety” (5 measures of traffic-related measures), “street amenity” (3 measures for having shady trees, street lamps, and shops/businesses), and “upkeep” (3 measures for street cleanliness, surface maintenance, and upkeep of buildings and lots).

### 2.4. Analysis

The walkability measures were aggregated at the ZIP level (*n* = 38) for this study, as only ZIP information of the participants' residences was available. The aggregation ranged from 1 to 7 street segments per ZIP area, with a mean of 3 street segments per ZIP area. A series of multivariate logistic regression analyses were conducted to obtain the odds ratios and 95% confidence intervals of walking behavior (any walking and recommended-level walking) by measures of walkability, while controlling for age, gender, race/ethnicity, education, BMI, physical impairment, and social support for exercise. LOGISTIC procedure of SPSS PC Version 20 (IBM Corporation) was used. All analyses were completed in 2013.

## 3. Results and Discussion

As shown in Table 2, the average age of participants was 62 years, and 61.1% were female. Non-Hispanic Whites were the largest racial/ethnic group (56.7%), followed by non-Hispanic Blacks (35.1%). The mean BMI was 32.0 kg/m^2^ (SD 6.9), and 63.0% had an impairment that affected physical activity, with a muscular/skeletal impairment as the most prevalent type (45.7%). Social support score had a mean of 2.35 (SD 1.60). About half of participants (51.9%) reported that they walked for exercise on community streets. Those who engaged in the recommended level of walking (≥150 minutes/week) represented 29.8% of the entire sample. The overall walkability scores of the respondents' communities at the ZIP level were 2.39 (SD 0.57), and subgroup walkability scores ranged from 2.11 (SD 0.73) for street amenity to 3.09 (SD 0.37) for upkeep.

Table 3 represents the results of logistic regression analyses. Any walking was associated with traffic safety (OR 1.34, 95% CI 1.15–1.65), but it was not significantly associated with overall walkability. Recommended-level walking was associated with overall walkability (OR 2.65, 95% CI 1.22, 5.74), as well as sidewalks (OR 1.73, 95% CI 1.12–2.67), street amenity (OR 2.04, 95% CI 1.12–3.71), and traffic safety (OR 1.92, 95% CI 1.02–3.72).

These findings are generally congruent with existing studies that evaluate the association between perceived walkability and exercise walking among adults with diabetes. Deshpande et al. reported that the perceived availability of many nearby places to walk, shoulders on nearby streets, and rating of the community as generally pleasant were environmental factors associated with regular physical activity among rural adults with diabetes [24]. Taylor et al. found that self-reports of having connective streets, shops, a transit stop nearby, and interesting things to look at were street factors associated with walking for leisure among adults with diabetes in a Canadian city [25]. de Greef et al. found that perceived walkability and aesthetics of the neighborhood were among the most consistent street environmental correlates with physical activity in adults with diabetes in Belgium [26].

The present study, however, found that upkeep, the measure that is related to aesthetics and pleasantness, was not associated with walking for exercise. Traffic safety was significantly associated with two levels of walking in the present study, but the existing perception-based studies did not find traffic safety as a significant correlate. It has been found that perceived and objectively measured environments do not necessarily correlate, and they could also relate differently to physical activity [28]. Literature indicates that discrepancies between perceived and objective environments are wider among individuals of low socioeconomic status [35], the less physically active [36], and the elderly [37], the segments of population that are likely to be affected by diabetes. Further research is needed to investigate relative importance of perceived and objective built environments in predicting walking behavior among adults with diabetes.

The recommended level walking's relatively strong association with overall walkability (OR 2.65) suggests salience of the built environment in adults with diabetes' decision to substantially engage in this activity. The robust result can also be attributed to having geographically large study communities with a wide range of environmental features as well as sociodemographically diverse study participants. In addition, it was found that among the covariates entered in the logistic regression analyses, social support was significantly associated with any walking (OR 1.24, 95% CI 1.03, 1.49) as well as the recommended level walking (OR 1.27, 95% CI 1.04, 1.53) (data not shown). This finding is consistent with existing literature, where social support was found to be an important determinant of physical activity [38, 39].

Limitations exist in this study. The measures of walkability were aggregated at the ZIP level due to the lack of street address information of the participants. The walkability measures in this study should be interpreted as the community-level walkability and not the street-level walkability. The walking behavior measures were derived from the validity and reliability-tested BRFSS instruments, but they could be affected by recall and/or social-desirability bias. The analyses were not adjusted for seasonality, which could affect outdoor walking behavior. Self-selection into walkable areas was not controlled in the analysis. Finally, cross-sectional design did not allow us to examine a causal relationship between walkability and walking behavior.

## 4. Conclusions

This study examined the association between objectively measured walkability and walking for exercise among adults with diabetes and found that any walking for exercise on community streets was associated with traffic safety, while exercise walking at the level recommended by diabetes experts for achieving glycemic control was associated with overall walkability as well as sidewalks, street amenities, and traffic safety. All these analyses were controlled for age, gender, race/ethnicity, education, BMI, physical impairment, and social support for exercise.

The findings of this study provide important insights into public health practice. Community walking is a popular, inexpensive, convenient, and potentially sustainable physical activity that can bring many health benefits to people with diabetes. As this study suggests, traffic safety features, well-designed sidewalks, and street amenities are the objective built environment elements that can support the expert-recommended level of walking. Public health professionals who plan to implement a community walking program for adults with diabetes can enhance program effectiveness by evaluating these key features in the community and carefully planning program settings and walking routes to make the best out of the existing built environment. If resources are available, structural barriers can be modified and new supportive features can be added. Along with individual-level health promotion and social support, walkability of the community should be an integral part of the comprehensive socioecologic approach to increase physical activity in adults with diabetes.

## Figures and Tables

**Table 1:** Individual walkability measure scoring system for the Walking Environment Audit Tool-Diabetes (WEAT-D).

Item	Points			
	4	3	2	1
Sidewalk*				
Sidewalk—coverage of the street	All along	Partial (≥50%)	Partial (<50%)	None
Material of sidewalk	Concrete	Asphalt	Brick	Loose pebbles/dirt
Width of sidewalk	≥72′′	54–71′′	36–53′′	<36′′
Buffer—% coverage of the sidewalk	100%	75–99%	50–74%	>50%
Material of buffer	Grass	Concrete or asphalt	Brick	Loose pebbles/dirt
Width of buffer	≥72′′	54–71′′	36–53′′	<36′′
Curb—% coverage of the sidewalk	100%	75–99%	50–74%	>50%
Height of curb	≥6′′	4.5–5.9′′	3.0–4.4′′	>3.0′′
Sidewalk curb ramp	Smooth, no gap	Small gap	Major gap	No ramp
Traffic safety				
Street lane type	One-way single	One-way double	Two-way, single each	Two-way, double each
Speed limit	<30 MPH	30–39 MPH	40–49 MPH	≥50 MPH
Volume of car traffic	Little or no	Light	Moderate	Heavy
Signal	All-time pedestrian	Push-button pedestrian	Traffic only	None
Cross walk	Clearly marked	Partially faded	Mostly faded	None
Street amenity				
Street lights	All along	Partial (≥50%)	Partial (<50%)	None
Shady trees	All along	Partial (≥50%)	Partial (<50%)	None
Shops and businesses	>30%	15–29%	1–14%	0%
Upkeep				
Surface cleanliness	Excellent	Good	Fair	Poor
Surface structural maintenance	Excellent	Good	Fair	Poor
Well-kept buildings and lots	100%	85–99%	70–84%	<70%

*1 point is given to each subcategory if there is no sidewalk.

**Table 2:** Sociodemographics, health status, physical activity, and community walkability of the study population.

Characteristic	Total sample (*n* = 208)
Age, mean (SD)	62.0 (14.7)
Female, *n* (%)	127 (61.1)
Race/ethnicity, *n* (%)	
Non-Hispanic White	118 (56.7)
Non-Hispanic Black	73 (35.1)
Hispanic	5 (2.4)
American Indian	4 (1.9)
Other	8 (3.8)
Education, *n* (%)	
Less than high school	51 (24.5)
High school graduate	70 (33.7)
Some college or higher	87 (41.8)
Body mass index, mean (SD)	32.0 (6.9)
Having any impairment that affects physical activity, *n* (%)	131 (63.0)
Type of impairment (multiple responses), *n* (%)	
Muscular/skeletal	95 (45.7)
Pulmonary	18 (8.7)
Neurological	11 (5.3)
Cardiovascular	11 (5.3)
Vision/hearing	10 (4.8)
Other	25 (12.0)
Social support for exercise, mean (SD)*	2.35 (1.60)
Physical activity in the previous month, *n* (%)	
Walked in the community for exercise, any minutes/week	108 (51.9)
Walked in the community for exercise, ≥150 min/week	62 (29.8)
Community's walkability measures, mean (SD)^†^	
Overall walkability	2.39 (0.57)
Sidewalks	2.31 (0.99)
Traffic safety	2.31 (0.28)
Street amenity	2.11 (0.73)
Upkeep	3.09 (0.37)

*A composite variable for having a friend, spouse, or child exercises with you. 1: not at all; 5: great deal.

^†^Summary measures of walkability in the 1–4 scale, with 4 as the most desirable feature.

**Table 3:** Relationship between walkability measures and walking for exercise in the community among adults with diabetes.

Objectively measured walkability	Any walking		Walking ≥150 min/week	
	OR (95% CI)	*P* value	OR (95% CI)	*P* value
Overall walkability	1.75 (0.92, 3.35)	0.090	2.65 (1.22, 5.74)	0.014
Subcategory				
Sidewalks	1.29 (0.88, 1.88)	0.198	1.73 (1.12, 2.67)	0.013
Traffic safety	1.34 (1.15, 1.65)	0.011	1.92 (1.02, 3.72)	0.047
Street amenity	1.24 (0.74, 2.06)	0.415	2.04 (1.12, 3.71)	0.020
Upkeep	0.75 (0.28, 2.05)	0.580	0.36 (0.12, 1.06)	0.630

Controlling for age, sex, race, education, body mass index, physical impairment, and social support for exercise.
